# The Rad4^TopBP1^ ATR-Activation Domain Functions in G1/S Phase in a Chromatin-Dependent Manner

**DOI:** 10.1371/journal.pgen.1002801

**Published:** 2012-06-28

**Authors:** Su-Jiun Lin, Christopher P. Wardlaw, Takashi Morishita, Izumi Miyabe, Charly Chahwan, Thomas Caspari, Ulrike Schmidt, Antony M. Carr, Valerie Garcia

**Affiliations:** 1 Genome Damage and Stability Centre, University of Sussex, Brighton, Sussex, United Kingdom; 2 Department of Molecular Genetics, University of Toronto, Toronto, Ontario, Canada; 3 School of Biological Sciences, Bangor University, Bangor, Gwynedd, United Kingdom; Duke University, United States of America

## Abstract

DNA damage checkpoint activation can be subdivided in two steps: initial activation and signal amplification. The events distinguishing these two phases and their genetic determinants remain obscure. TopBP1, a mediator protein containing multiple BRCT domains, binds to and activates the ATR/ATRIP complex through its ATR-Activation Domain (AAD). We show that *Schizosaccharomyces pombe* Rad4^TopBP1^ AAD–defective strains are DNA damage sensitive during G1/S-phase, but not during G2. Using *lacO*-LacI tethering, we developed a DNA damage–independent assay for checkpoint activation that is Rad4^TopBP1^ AAD–dependent. In this assay, checkpoint activation requires histone H2A phosphorylation, the interaction between TopBP1 and the 9-1-1 complex, and is mediated by the phospho-binding activity of Crb2^53BP1^. Consistent with a model where Rad4^TopBP1^ AAD–dependent checkpoint activation is ssDNA/RPA–independent and functions to amplify otherwise weak checkpoint signals, we demonstrate that the Rad4^TopBP1^ AAD is important for Chk1 phosphorylation when resection is limited in G2 by ablation of the resecting nuclease, Exo1. We also show that the Rad4^TopBP1^ AAD acts additively with a Rad9 AAD in G1/S phase but not G2. We propose that AAD–dependent Rad3^ATR^ checkpoint amplification is particularly important when DNA resection is limiting. In *S. pombe*, this manifests in G1/S phase and relies on protein–chromatin interactions.

## Author Summary

DNA structure–dependent checkpoint activation and the amplification of checkpoint signals are carefully modulated to allow the checkpoint kinases to delay mitosis and regulate DNA metabolism. While much work has gone into understanding how this checkpoint functions, the mechanism by which the checkpoint signal is amplified is less clear. We have characterised a conserved domain in the *Schizosaccharomyces pombe* TopBP1 homolog, Rad4^TopBP1^ (also known as Cut5) that is capable of activating the ATR homolog Rad3^ATR^. We demonstrate that this domain is not required for initial checkpoint activation, but functions to amplify the checkpoint signal, likely when the presence of single-stranded DNA is limiting. Our data suggest that the function of the Rad4^TopBP1^ ATR-Activation Domain (AAD) is mediated by interactions between checkpoint proteins and phosphorylated histone H2A, which is itself promoted by Rad3^ATR^. We propose that the resulting amplification of the checkpoint signal is particularly important in G1-S phase, when resection is limited.

## Introduction

The DNA damage checkpoint is an elaborate signal transduction pathway that monitors the integrity of the DNA, prevents cell cycle progression and promotes appropriate DNA metabolism [1] reviewed in [2]. The DNA damage sensors associated with checkpoint activation define two separate DNA structure-dependent signal transduction cascades. Each pathway engages a phospho-inositol-3 kinase-like protein kinase (PIKK); either the Ataxia Telangiectasia Mutated (ATM) or the Ataxia Telangiectasia and Rad3 related (ATR) kinase [3]. ATM detects DNA double strand breaks (DSBs) by interaction with the Mre11-Rad50-Nbs1 repair complex, while ATR primarily senses single stranded-DNA (ss-DNA) through interactions with RPA. Both ATM and ATR are conserved in the model organisms *S. pombe and S. cerevisiae*.

**Figure 1:**
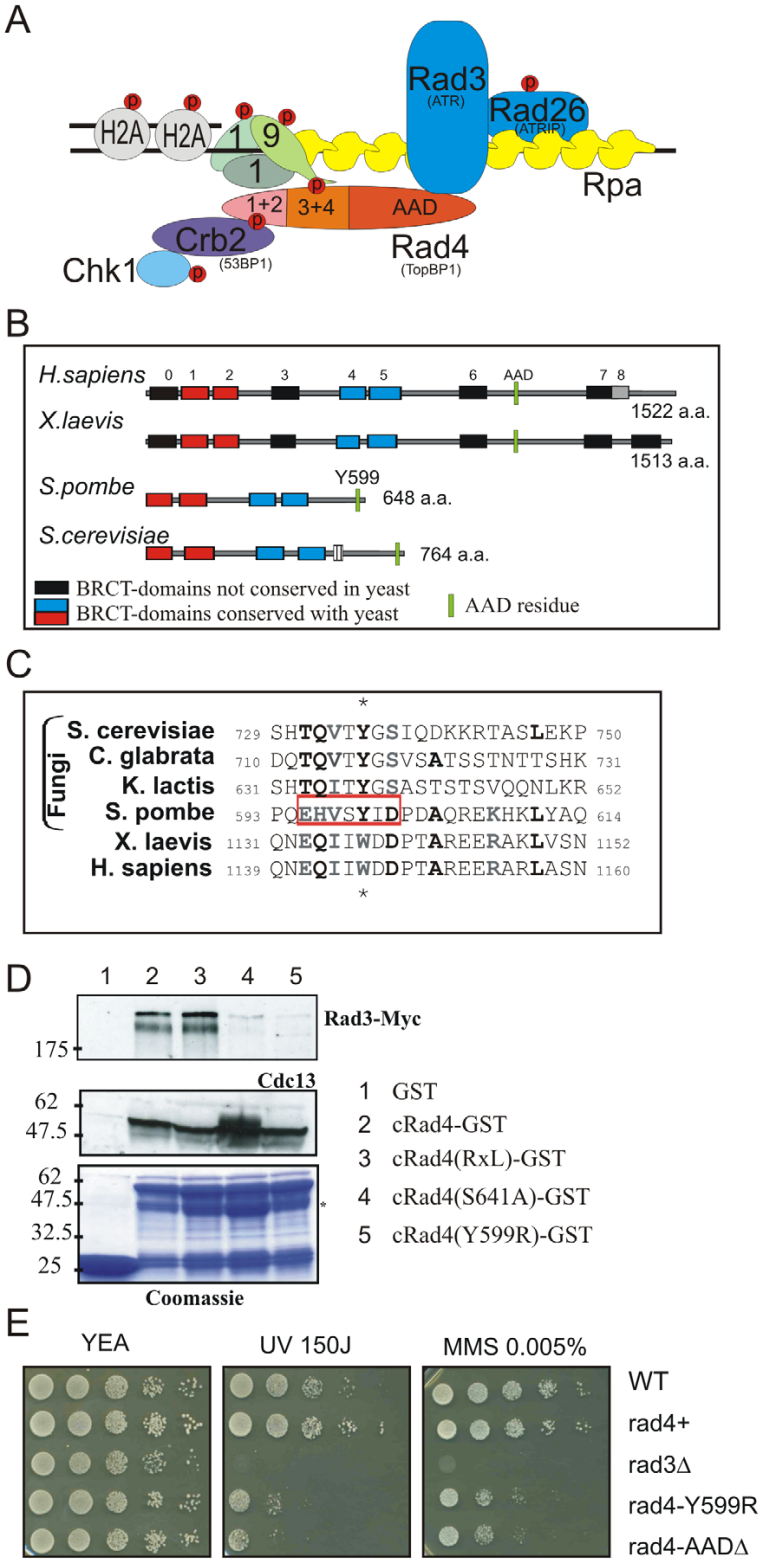
Conservation of the Rad4^TopBP1^ ATR-Activation Domain. A. Cartoon showing the interactions of the fission yeast homolog of TopBP1 (Rad4) during checkpoint activation. See text for details. B. Schematic representation of TopBP1 homologs. BRCT domains are boxed. Those conserved with the yeast homologs are shown in blue (1+2) and red (4+5). The position of the conserved AAD aromatic residue is indicated in green. C. Corresponding amino acid sequence alignment. The region deleted in *rad4-Δ[595–601]* is boxed. Conserved residues are in bold and the aromatic residue starred. D. Recombinant GST and GST-fusion proteins were used for affinity capture from soluble *myc-rad3 S. pombe* extracts. Cdc13^CyclinB^ was detected with α-Cdc13, Rad3^ATR^ with α-Myc. E. Spot-test analysis of UV and MMS sensitivity for the indicated strains. Serial dilutions of logarithmically growing cells were plated onto YE plates containing the indicated drugs. *rad4* ^+^ indicates the RMCE control strain (*rad4* ^+^ locus flanked by loxP and loxM sites, see reference 56) and acts as a control for the defined *rad4* alleles (where the same arrangement of lox sites flank the mutated gene at the *rad4* locus).

For ATR to recognise a DNA lesion, single-stranded DNA (ssDNA) needs to be formed - for example by DNA repair-dependent DNA processing [4] or following the replication machinery encountering the unrepaired lesion [5]. Once ssDNA is generated, it is immediately coated by replication protein A (RPA) (Reviewed in: [6]). Multiple ATR molecules are initially recruited to ssDNA regions via ATRs obligate binding partner, ATRIP, which binds directly to RPA [7], [8]. ATR-ATRIP recruitment to ssDNA-RPA is necessary for “basal” ATR activation, but is insufficient for full checkpoint activation: co-recruitment of a second complex consisting of three PCNA-like proteins, Rad9, Hus1 and Rad1 (known as the 9-1-1 clamp) is also necessary. 9-1-1 is loaded in parallel to ATR recruitment at 5′ ssDNA/dsDNA junctions by the checkpoint clamp loader Rad17-RFC_[2–5]_ [9], [10], [11]. (Figure 1A).

When ATR-ATRIP is first loaded at the site of ssDNA, its “basal” kinase activity promotes phosphorylation of its immediate neighbours, including ATRIP [12], [13], an in trans phosphorylation of a residue within ATR itself, T1989 [14], and the subunits of the 9-1-1 clamp [15], [16]. Dependent on the concomitant recruitment of 9-1-1, a further protein, TopBP1, is recruited [17]. TopBP1 is recruited via an interaction between its BRCT (1+2) domains and a constitutive phosphosphorylation on the C-terminus of Rad9 [18], [19]. Similarly in both yeast systems, *Saccharomyces cerevisiae* and *Schizosaccharomyces pombe*, the TopBP1 homologs, Dpb11 and Rad4 respectively, are recruited by the phosphorylation of the C-terminus of Rad9^Ddc1^ creating a binding site for a pair of BRCT domains (Figure 1A). In *S. pombe*, the C-terminal phosphorylations occur on Rad9 at residues T412 and S423 [20]. This subsequently recruits Rad4^TopBP1^ via interaction with BRCT pair (3+4). However, unlike in mammalian cells, T412 and S423 in *S. pombe* are directly targeted by Rad3^ATR^ in response to its ssDNA/RPA binding and concomitant 9-1-1 loading [16], [20], [21]. Despite these differences, in both *S. pombe* [20] and mammalian cells [14], Rad4^TopBP1^ recruitment promotes the formation of a Rad3^ATR^/9-1-1/Rad4^TopBP1^ complex (Figure 1A). However, the mode of interaction of Rad3^ATR^ and Rad4^TopBP1^ within this complex has not been defined in *S. pombe*.

Mammalian TopBP1 can directly activate ATR-ATRIP both in vitro in the absence of ssDNA/RPA and when over-expressed in cells. TopBP1-dependent ATR activation requires an ATR activation domain (AAD) situated between the 6^th^ and 7^th^ BRCT domains [17] and mutation of a conserved aromatic residue within this unstructured region, W1147, prevents this mode of ATR activation. The AAD contacts a region within the C-terminus of ATR, between the kinase and FATC domains [22], which has been termed the PIKK Regulatory Domain (PRD). Mutation of a conserved PRD residue, K2598, similarly abolishes TopBP1-dependent ATR activation. In both mammalian cells and Xenopus extracts the interaction between TopBP1 and ATR-ATRIP appears to be essential for checkpoint activation in response to replication stress [17], [22], although the initial *in trans* phosphorylation of ATR on T1989, reportedly essential for full ATR activation, is TopBP1-independent [14].

In the budding yeast model system, the intrinsically disordered C-terminal extension of the TopBP1 homolog, Dpb11^TopBP1^, contains an AAD which interacts with and activates Mec1^ATR^ via a pair of aromatic residues, W700 and Y735 [22], [23], [24], [25]. Interestingly, in *S. cerevisiae*, at least two distinct Mec1^ATR^ activation domains have been identified: in addition to the Dbp11^TopBP1^ AAD, the C-terminal tail of Ddc1^Rad9^ (*S. cerevisiae* homolog of the 9-1-1 subunit Rad9) contains an AAD that can directly stimulate Mec1^ATR^ activity in vitro and contributes to checkpoint activation in vivo [11], [26]. The key residues in Ddc1^Rad9^ required for Mec1^ATR^ activation are W352 and W544. W352 resides on the surface of the PCNA-like domain, while W544 lies within the intrinsically disordered C-terminus. In vivo the Ddc1^Rad9^ AAD is essential for Mec1^ATR^ activation when *S. cerevisiae* cells are in G1 [24], [26], while the Ddc1^Rad9^ AAD acts redundantly with the C-terminal AAD of Dpb11^TopBP1^ during checkpoint activation in G2. It is proposed that, at least in *S. cerevisiae*, a minimum of one other protein contains an equivalent AAD (Reviewed in [27]).

We have previously shown that *S. pombe* Rad4^TopBP1^ is not required for the Rad3^ATR^-dependent and DNA damage-dependent phosphorylation of Rad26^ATRIP^ or the 9-1-1 clamp subunits [16], [20], demonstrating that Rad3^ATR^ is active at sites of DNA damage in the absence of activation by the Rad4^TopBP1^ AAD. However, the presence of Rad4^TopBP1^ is clearly required to form a robust Rad3^ATR^/9-1-1/Rad4^TopBP1^ complex [20], to recruit the Crb2^53BP1^ mediator protein [28], [29] and for Rad3^ATR^ to phosphorylate downstream substrates such as Chk1-S345 [30] and promote robust checkpoint activation.

To further explore the role of Rad4^TopBP1^ in checkpoint activation in *S. pombe* we identified and characterised the Rad4^TopBP1^ AAD. We show that Rad4^TopBP1^ can interact with Rad3^ATR^ via its AAD and that the AAD contributes to Rad3^ATR^ activation in vivo. We observe that the biological function of the Rad4^TopBP1^ AAD is most important in G1/S phase, when resection is limited, and that reducing DSB resection in G2 following ionising radiation results in compromised Chk1 phosphorylation in the absence of Rad4^TopBP1^ AAD function. In order to separate out and study Rad4^TopBP1^ AAD-dependent Rad3^ATR^ activation we developed a Rad4^TopBP1^ AAD-dependent *lacO*-LacI checkpoint activation system for *S. pombe* and used this to show that Rad4^TopBP1^ AAD-dependent Rad3^ATR^ activation is also dependent on histone H2A phosphorylation. Consistent with a role for this chromatin modification, mutations in Crb2 that interfere with phospho-binding by Crb2 also decrease Rad4^TopBP1^ AAD-dependent checkpoint activation. Thus, the Rad4^TopBP1^ AAD-dependent Rad3^ATR^ activation pathway is chromatin dependent, implying a role in checkpoint amplification and maintenance.

## Results

In *S. cerevisiae*, two aromatic residues, W700 and Y735 were identified as critical for Dbp11^TopBP1^ AAD activity [24], [25]. We similarly created an alignment of Rad4^TopBP1^ with the C-terminal tails of a variety of TopBP1 homologs and identified a short sequence encompassing Y599 in *S. pombe* (Figure 1B, 1C), which we subsequently confirmed as defining the AAD activity of Rad4^TopBP1^ (see below). To characterise the function of the Rad4^TopBP1^ AAD, we separately mutated the conserved aromatic amino acid to create strain *rad4-Y599R* or deleted the minimal conserved motif to create *rad4-Δ[595–601]*.

To establish if the Rad4^TopBP1^ AAD interacts with Rad3^ATR^ we expressed and purified recombinant Rad4[288–648] (that includes BRCT (3+4) and the unstructured C-terminal tail) as a fusion with GST. Constructs containing a mutation in either the AAD (Y599R), deletion of residues 643–645 encompassing a putative cyclin binding motif (RxL), or mutation of a CDK phosphorylation site (S641A) were similarly purified. Wild-type and all the mutated recombinant proteins, when incubated with yeast extracts, bound to and co-purified the control interacting partner of Rad4^TopPB1^, Cdc13^CyclinB^ (unpublished data). Wild-type Rad4-GST also co-purified Rad3^ATR^-Myc from extracts prepared from *3myc-rad3* cells (Figure 1D). In contrast, the amount of Rad3^ATR^-Myc pulled-down with recombinant AAD-mutated Rad4^TopBP1^-GST was reproducibly reduced (n = 4). Neither Rad3^ATR^-myc nor Cdc13^CyclinB^ were pulled-down with a GST-only control. We conclude that, *in vitro*, this residue of Rad4^TopBP1^ is part of a Rad4^TopBP1^ interaction domain for Rad3^ATR^, consistent with it having the properties of an AAD. We also note that the interaction with Cdc13^CyclinB^ is not abolished by loss of the RxL motif and that mutation of S641 affects the Rad4^TopBP1^:Rad3^ATR^ interaction in vitro (Figure 1D, lane 4).

### *rad4-AAD* mutants show checkpoint defects associated with S phase

**Figure 2:**
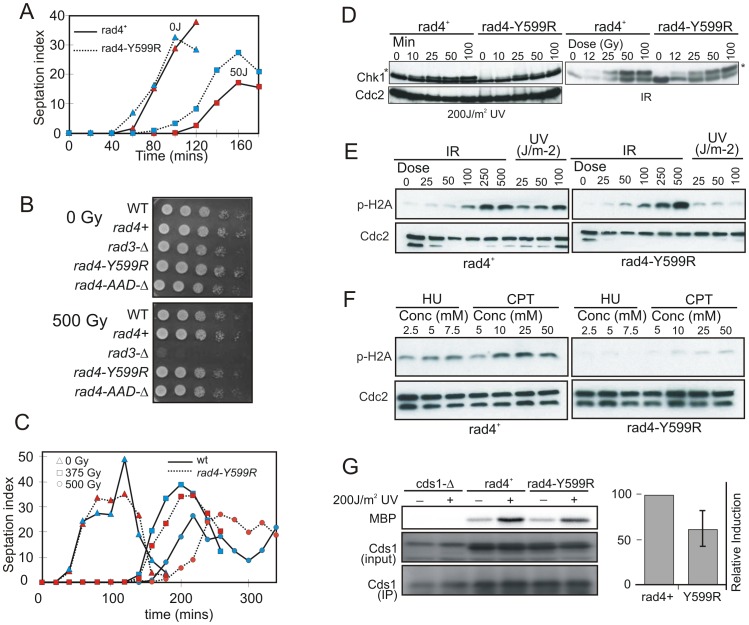
Checkpoint activation in response to genotoxic stress. A. *rad4* ^+^ and *rad4-Y559R* strains were synchronised in G2 by lactose gradient centrifugation, mock irradiated or irradiated with 50 Jm-2 of UV. Cell septation index, which reflects progression through mitosis, was observed to monitor G2 arrest. B. Spot-test analysis of IR sensitivity for the indicated strains. *rad4* ^+^ indicates the RMCE control strain (*rad4* ^+^ locus flanked by loxP and loxM sites). C. *rad4* ^+^ and *rad4-Y559R* cells were synchronised in G2 by lactose gradient centrifugation, mock irradiated or irradiated with either 375 Gy or 500 Gy IR. Cell septation index was observed to monitor G2 arrest. D. Western blot analysis over time of HA-Chk1, detected with α-HA, in response the indicated dose of UV or IR in *rad4* ^+^ and *rad4*-Y599R (AAD-defective). * indicates phospho Chk1. E. Analysis of γH2A induction after the indicated doses of IR and UV treatment (20 minutes after irradiation). γH2A is detected with α-pS129 Ab. F. Equivalent analysis for γH2A after cell growth in the indicated doses of HU (1 hour) and CPT (30 mins). G. Cds1^Chk2^ kinase activity. Cds1 was immuno-precipitated from *rad4* ^+^ or *rad4-Y559R* strains with α-Cds1 one hour after UV irradiation and equivalent amounts of protein tested for kinase activity against MBP. Note the non-specific band in the input, which, following IP, does not resolve from Cds1. The experiment was repeated three times and the relative induction for rad4-Y599R compared to wild type is shown on the right. *rad4* ^+^ indicates the RMCE [56] control strain.

Both *rad4-Y599R* and *rad4-Δ[595–601]* displayed normal cell cycle progression (data not shown), indicating that, as is observed in *S. cerevisiae* [24], the Rad4^TopBP1^ AAD is not required for unperturbed DNA replication. In response to UV, MMS and HU treatment, *rad4-Y599R* and *rad4-Δ[595–601]* cells showed intermediate sensitivity when compared to *rad4* ^+^ and checkpoint defective *rad3*Δ (Figure 1E and Figure S1A). To establish if the sensitivity to DNA damage correlated with a defective G2 DNA damage checkpoint, we monitored cell cycle progression after cells were synchronised in G2 and UV-irradiated. Following exposure to 50 Jm-2 (Figure 2A), *rad4-Y599R* cells displayed premature release from cell cycle arrest (∼20 min earlier than *rad4* ^+^ after 50 Jm-2). We next monitored the sensitivity and checkpoint response to ionising radiation. *rad4-Y599R* mutant cells displayed only very mild sensitivity to IR (Figure 2B) and the checkpoint was mildly extended (Figure 2C). We do not know the reason for the slight extension of the G2 delay: no obvious increase in numbers or duration of Rad22^Rad52^ foci were observed, indicating no significant delay to DSB repair (Figure S1B).

We next examined Chk1 phosphorylation status in *rad4* ^+^ and *rad4-Y599R* cells as a surrogate for checkpoint activation (Figure 2D). In response to 200 Jm-2 UV irradiation, asynchronously growing *rad4-Y599R* cells displayed reduced Chk1 phosphorylation when compared to *rad4* ^+^ cells, consistent with the partial checkpoint defect observed. Conversely, in response to IR, no significant difference is seen between *rad4-Y599R* and *rad4* ^+^. An upstream target of Rad3^ATR^ is the C-terminus of histone H2A [31], [32]. To establish if the UV-specific defect in Rad3^ATR^-dependent phosphorylation is specific to Chk1, we monitored γH2A formation following either UV or IR treatment (Figure 2E). As was seen for Chk1 phosphorylation, a significant decrease in γH2A is observed in *rad4-Y599R* cells following UV but not IR treatment when compared to *rad4* ^+^ cells.

The pattern of DNA damage sensitivity seen for *rad4-Y599R* cells is consistent with a specific sensitivity within S phase. >70% of fission yeast cells in an asynchronous culture are in G2 and mitosis is followed rapidly by S phase: G1 is extremely short. In response to IR, the G2 DNA damage checkpoint is robustly activated and DNA repair completed before cells pass through mitosis and into S phase [33]. Thus, following IR, relatively few cells replicate damaged DNA. Conversely, the G2 checkpoint is not robustly activated following UV [34] and the majority of UV-irradiated cells pass through mitosis and enter S phase with damaged DNA. To monitor S phase-specific events, we thus examined γH2A induction in cells treated with either hydroxyurea (HU), an inhibitor of ribonucleotide reductase, or Camptothecin (CPT), an inhibitor of topisomerase I (Figure 2F). Consistent with both agents manifesting cytotoxicity in S phase, γH2A levels were significantly reduced when comparing *rad4-Y599R* with *rad4* ^+^ cells. Finally, since replication of UV damaged DNA induces Cds1^Chk2^ activity [35], we monitored the kinase activity of immuno-precipitated Cds1^Chk2^ following UV irradiation of *rad4-Y599R* and *rad4* ^+^ cells (Figure 2G). Cds1^Chk2^ activation was reproducibly lower for *rad4-Y599R*, indicating an impaired S phase checkpoint activation (n = 3).

### The *rad4-AAD* mutants are sensitive to DNA damage in S-phase

**Figure 3:**
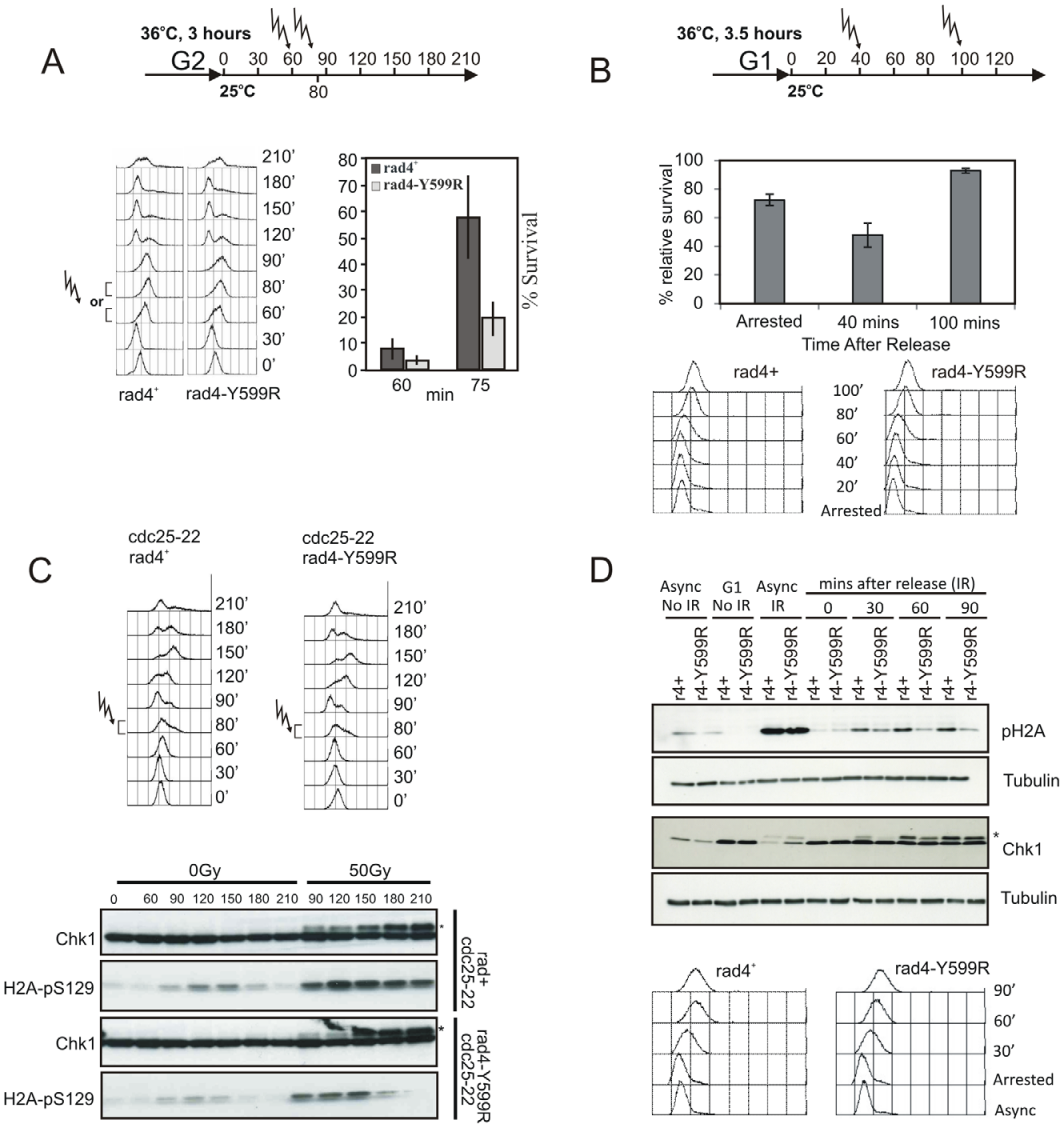
The Rad4^TopBP1^ AAD is defective in checkpoint activation in S phase. A. *rad4* ^+^ and *rad4*-*Y599R* mutant cells were synchronised in G2 by *cdc25* arrest and released into the cell cycle (experimental schematic: top). DNA content was assessed by FACS analysis (left) and cells were irradiated (50 Gy) either in mid or late S phase. Viability was assessed by colony formation (right). B. *cdc10-m17 rad4* ^+^ and *cdc10-m17 rad4-Y599R* cells were synchronised in G1 and exposed to 50 Gy IR either before, 40 min after or 100 min after release (G1/S, mid S, late S/G2). % cell survival of *rad4-Y599R* compared to WT is shown. Error bars: standard deviation (n = 3). C. An equivalent experiment was performed to assess phospho-Chk1 (*) and γH2A induction over time by western blot with α-HA or α-pS129 following 50 Gy IR. D. As an alternative synchronisation strategy, *rad4* ^+^ and *rad4-Y599R* mutant cells were synchronised in G1 by *cdc10-m17* arrest and released into S phase and irradiated (50 Gy). γH2A was monitored using α-pS129. Chk1 phosphorylation was monitored using α-HA. A representative FACS profile of untreated cultures is shown below. * indicates phospho Chk1.

If the *rad4-Y599R* mutant is deficient in activation of Rad3^ATR^ in S phase, we would anticipate increased sensitivity to IR within S phase when compared to *rad4* ^+^ cells. To test this possibility, *rad4-Y599R* mutant and *rad4* ^+^ cells where either synchronised in G2 cells using *cdc25-22* or in G1 using a *cdc10-m17*. Following the block, cells were released by reducing the temperature and cell cycle progression was monitored by FACS analysis (Figure 3A, 3B). Cells were irradiated with 50 Gy IR at the times indicated. *rad4-Y599R* cells showed significant increased sensitivity when compared to *rad4* ^+^ when irradiated in S phase, but not when irradiated in G2, when S phase is complete (i.e. see Figure 3B). In an equivalent *cdc25*-22 block and release experiment, we monitored Chk1 phosphorylation and γH2A induction (Figure 3C). Unlike when asynchronous *rad4-Y599R* cells are irradiated (>70% of such cells are in G2), when *rad4-Y599R* cells were irradiated in early-mid S phase, Chk1 phosphorylation was moderately reduced for the first 40 minutes after irradiation and γH2A levels were similarly decreased when compared to *rad4* ^+^. Interestingly, following progression through S phase and into G2 (150 minute time point), Chk1 phosphorylation levels increased significantly in *rad4-Y599R* cells, although the same was not seen for γH2A levels.

To determine that the use of *cdc25-22* synchronisation was not generating an artefact (Cdc25 is an activator of Cdc2-Cyclin B, which itself is required for normal DNA damage responses in G2 [36]), we used the alternative method of synchronisation where cells were arrested in G1 using *cdc10-M17* and released directly into S phase (Figure 3D). Unlike IR treatment of asynchronous cultures where equivalent levels of γH2A were observed (Async IR), treatment of *rad4-Y599R* cells at 30, 60 or 90 minutes after release from arrest resulted in decreased γH2A levels and Chk1 phosphorylation when compared to *rad4* ^+^ control cells.

### *LacO* array-dependent checkpoint activation in *S. pombe* requires the Rad4^TopBP1^ AAD

**Figure 4:**
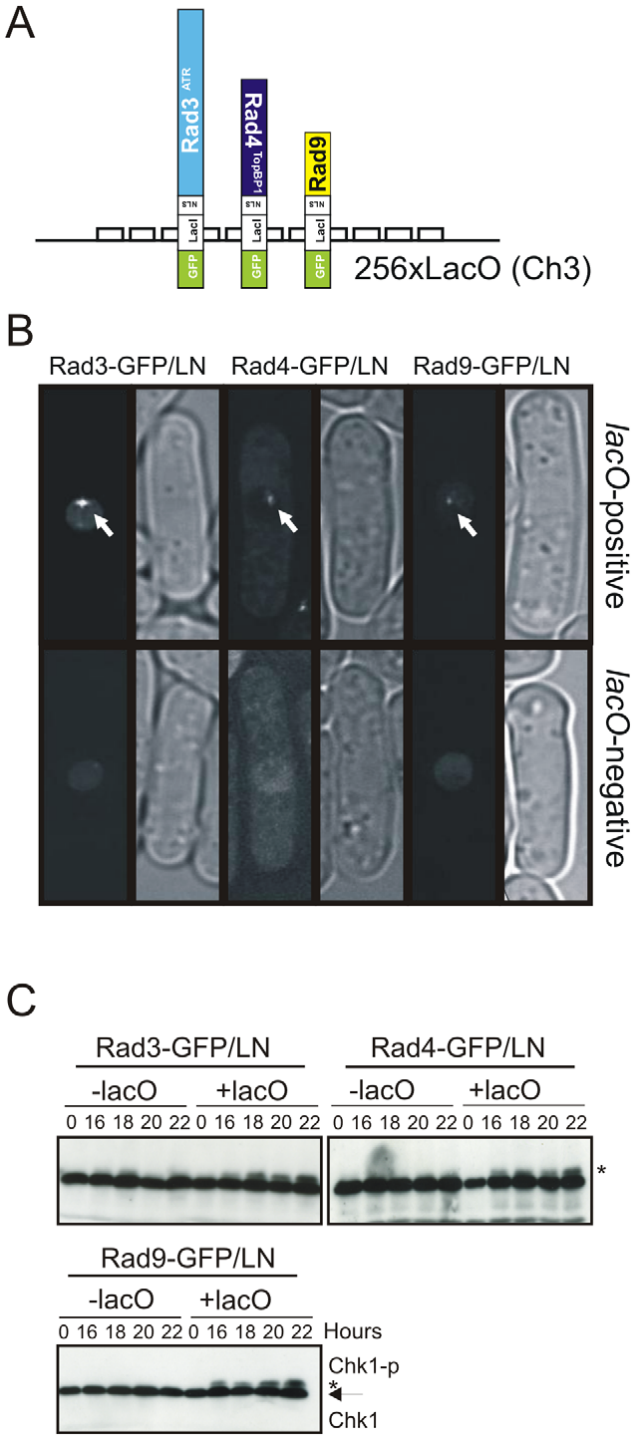
Checkpoint protein recruitment to chromatin causes checkpoint activation. A. Schematic of constructs used. B. Indicated constructs were expressed in either a strain containing 256 integrated *lacO* repeats at the *ura4* ^+^ locus (*lacO* positive) or in an isogenic control (*lacO* negative) and GFP visualised in fixed cells. Arrow indicates a visible foci (t = 24 hours). **C**. The phosphorylation status of Chk1 was detected with α-HA over 24 hours following induction of the indicated fusion construct in *lacO* negative or *lacO* positive cells by withdrawal of thiamine. * indicates phospho Chk1. Note that *nmt1* induction occurs approximately 14–18 hours after thiamine withdrawal.

In *S. cerevisiae*, co-localisation of two or more checkpoint proteins to arrays of *lacO* repeats bypasses the requirement for DNA damage in Mec1-mediated checkpoint activation [37]. To establish the role of the Rad4^TopBP1^ AAD in a what has previously been characterised as an RPA-ssDNA independent system, *rad4^TopBP1^*, *rad9* and *rad3^ATR^* were each fused to a construct encoding GFP, the *E. coli* lac-repressor (LacI) and a nuclear localization signal (NLS); GFP/LN (Figure 4A). The resulting plasmids express the fusion construct under the control of a thiamine-repressible (*nmt41*) promoter. We established that each of the fusion constructs were functional by expressing them individually in the corresponding null mutants. Each was able to suppress the DNA damage sensitivity (and for *rad4^TopBP1^*, the thermosensitivity) of the appropriate mutant, although for *rad4-GFP/LN* genotoxin resistance was not restored to wild-type levels (Figure S1C–S1E). When expressed in cells harbouring 256 repeats of the *lac* operator sequence (*lacO*) integrated at the *ura4* ^+^ locus, each fusion protein formed a single nuclear focus. No foci were detected in cells devoid of *lacO* arrays (Figure 4B).

**Figure 5:**
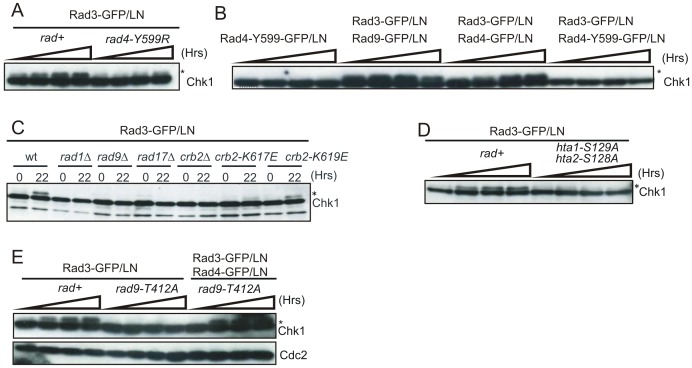
Genetic requirements for *lacO*-dependent Chk1 phosphorylation. * indicates phospho Chk1. A. The phosphorylation status of Chk1 was detected with α-HA over 24 hours following induction of Rad3-GFP/LN in *lacO* positive cells in the genetic background indicated. Increased time is indicated by the wedge. B. Equivalent experiments assessing Chk1 phosphorylation in *rad* ^+^ *lacO* positive cells upon induction of the indicated constructs. C. Chk1 phosphorylation was assessed upon expression of Rad3-GFP/LN in the indicated genetic backgrounds at t = 22 hours. D. The phosphorylation status of Chk1 was assessed following induction of Rad3-GFP/LN in *lacO* positive cells in *rad* ^+^ and the indicated double mutant where H2A cannot be phosphorylated. E. The indicated constructs were expressed in *rad* ^+^ or *rad9-T412* cells and Chk1 phosphorylation monitored. Rad9 T412 phosphorylation is required for the interaction of 9-1-1 complex with Rad4^TopBP1^.

We used Chk1 phosphorylation as a readout for DNA damage checkpoint activation (Figure 4C). Following thiamine removal (induction takes between 12 and 16 hours [38]), Chk1 became phosphorylated in *lacO* containing cells, but not in *lacO*-negative control cells, when either Rad3^ATR^, Rad4^TopBP1^ or Rad9 LacI fusion proteins were expressed. Similar results were obtained when each pair-wise combinations of two fusion proteins were expressed (Figure 5B). In *S. pombe*, DNA damage checkpoint activation results in cell cycle arrest and cell elongation. Elongated cells were observed upon expression of single fusion proteins (data not shown), confirming checkpoint activation. From these data we conclude that, in *S. pombe*, as in mammals [39] tethering of any of these single checkpoint proteins to a *lacO* array is sufficient to activate the DNA damage checkpoint and that, in contrast to the analogous experiments reported for *S. cerevisiae*, forced co-localisation of two checkpoint proteins is not required [37].

To establish if the Rad4^TopBP1^ AAD is involved in this damage-independent mode of checkpoint activation, we tested if Rad3^ATR^ tethering could result in Chk1 phosphorylation in a *rad4-Y599R* mutant background (Figure 5A). While Rad3-GFP/LN expression resulted in induced Chk1 phosphorylation in *rad4* ^+^ cells, Rad3-GFP/LN expression did not increase Chk1 phosphorylation in *rad4-Y599R* cells, demonstrating a role for the Rad4^TopBP1^ AAD. Next we established if expression and tethering of the AAD-defective Rad4-Y599R protein to *lacO* arrays was able to activate the checkpoint (Figure 5B). No induction of Chk1 phosphorylation was observed. Furthermore, while co-expression and tethering Rad3^ATR^ and Rad9, or of Rad3^ATR^ and Rad4^TopBP1^ resulted in checkpoint activation (Figure 5B), we observed that co-expression of Rad3^ATR^ with Rad4^TopBP1^-Y599R mutant protein did not result in Chk1 phosphorylation. This data suggests that the AAD-defective mutant protein can act as a dominant negative, at least in this specific situation, preventing the endogenous wild-type Rad4^TopBP1^ from functioning with the tethered Rad3^ATR^ to activate the checkpoint. It also supports the idea that the Rad4^TopBP1^ AAD domain is required for the activation of Rad3^ATR^ and not simply recruiting it.

### H2A phosphorylation is required for Rad4^TopBP1^ AAD–dependent checkpoint activation

While Rad3^ATR^ kinase activity is essential for Chk1 phosphorylation in response to DNA damage [40], it also depends on the 9-1-1 clamp, the Rad17 clamp loader and the Crb2^53BP1^ mediator. To characterise the dependencies for *lacO*-dependent checkpoint activation we examined which checkpoint genes were required for Chk1 phosphorylation during Rad3^ATR^ tethering (Figure 5C). Rad3^ATR^-GFP/LN was expressed in *lacO*-positive strains deleted for *rad1*, *rad9* (encoding 9-1-1 components), *rad17* (clamp loader) and *crb2*. Each was required for Chk1 phosphorylation. Thus, Rad3^ATR^ tethering is not sufficient for checkpoint activation: the clamp loader, the 9-1-1 clamp complex and the Crb2 mediator are all required and this artificial checkpoint activation system does not bypass the usual requirements. However, Brc1, the proposed MDC1/PTIP ortholog is not required for Chk1 phosphorylation in this system (Figure S1F)

In both *S. pombe* and *S. cerevisiae* recruitment of the 53BP1 ortholog (Crb2 and Rad9 respectively) to chromatin in response to IR requires prior phosphorylation of histone H2A [32], [41], [42]. In addition to H2A phosphorylation, recruitment also requires the largely constitutive methylation of a further histone residue, H3K79 in *S. cerevisiae* or H4K20 in *S. pombe*. These modifications are effected by distinct methylransferases in the two yeasts: Dot1 methylates H3K79 in *S. cerevisiae* [43] while Set9 methylates H3K20 in *S. pombe* [44], [45]. In *S. pombe* it has been demonstrated that the C-terminal BRCT domains of Crb2^53BP1^ binds directly to γH2A [42] while the Tudor domain binds directly to di-methylated H3K20 [46]. Both interactions are required for Crb2^53BP1^ chromatin association and show an epistatic relationship [45].

Using our Chk1 phosphorylation assay in response to Rad3^ATR^ tethering, we tested two strains harbouring charge reversal mutations of residues within the phospho-acceptor site of the C-terminal Crb2^53BP1^ BRCT domains, *crb2-K617E* and *crb2-K619E* (Figure 5C) that disrupt the interaction with γH2A [42]. Chk1 phosphorylation was reduced in both mutants. We next established if checkpoint activation by Rad3^ATR^ tethering was affected in cells containing mutants in the two H2A genes that replace the phosphorylated residue with alanine, *hta1-S129A hta2-S128A* [32]. Chk1 phosphorylation was not observed in this background (Figure 5D), indicating that the Rad3^ATR^ tethering-dependent and Rad4^TopBP1^ AAD-dependent checkpoint activation acts in the context of chromatin modification.

### Rad4^TopBP1^ tethering bypasses the requirement for Rad9 C-terminal phosphorylation

Upon activation of the DNA damage checkpoint in *S. pombe*, Rad3^ATR^ phosphorylates the Rad9 C-terminus on T412 and this is required to recruit Rad4^TopBP1^ [20]. Recruitment of Rad4^TopBP1^ allows subsequent recruitment of Crb2^53BP1^ and consequent Chk1 activation [29]. A similar requirement for TopBP1 recruitment via Rad9 C-terminal phosphorylation is also evident in *S. cerevisiae* and mammalian cells [19], [47], [48]. As expected, expression of Rad3^ATR^-LacI in cells harbouring a *rad9-T412A* mutation did not result in Chk1 phosphorylation (Figure 5E) implying that *lacO*-recruited Rad3^ATR^ must phosphorylate endogenous Rad9 to promote Rad4^TopBP1^ recruitment to activate the checkpoint.

We reasoned that the requirement for Rad9-T412 phosphorylation during activation by Rad3^ATR^ tethering may solely be to bring the Rad4^TopBP1^ AAD into proximity of Rad3^ATR^. In this case, we should be able to bypass the requirement for Rad9-T412 phosphorylation specifically for checkpoint activation by Rad3^ATR^ tethering by recruiting both Rad3^ATR^ and Rad4^TopBP1^ at the same time. Indeed, Chk1 phosphorylation was restored when we co-expressed Rad3^ATR^-LacI and Rad4^TopBP1^-LacI in a *rad9-T412A* mutant background (Figure 5E). Since checkpoint activation by co-expression of Rad3^ATR^-LacI and Rad4^TopBP1^-LacI remains *lacO* dependent (Figure S1G), these data suggest that Rad4^TopBP1^ AAD can activate the Rad3^ATR^-dependent checkpoint cascade in the absence of the recruitment activity of the Rad9 C-terminal tail.

### The Rad4^TopBP1^ AAD is important when resection is limiting

We have shown that the Rad4^TopBP1^ AAD functions to protect cells from insult during S phase, but is not required for G2 checkpoint activation after IR. Further, we demonstrated that when *rad4-Y599R* (AAD-defective) mutant cells were synchronised in S phase, phosphorylation of both Chk1 and H2A in response to IR treatment was reduced when compared to *rad4* ^+^ cells. The increase in Cdc2-Cdc13^ClyclinB^ (CDK) activity as cells progress from S phase into G2 [49] is known to establish conditions conducive to HR by regulating factors required for DNA resection [36], [50], [51]. A consequence of this is that, in response to IR treatment but not in response to UV treatment, ssDNA RPA is predicted to be more prevalent in G2 cells when compared to G1/S phase cells. We thus predicted that reducing resection rates associated with IR treatment in G2 cells would create a dependency for full Chk1 phosphorylation on the Rad4^TopBP1^ AAD and thus that Chk1 phosphorylation would be reduced in *rad4-Y599R* strains compared to *rad4* ^+^ strains in response to an equal dose of IR.

**Figure 6:**
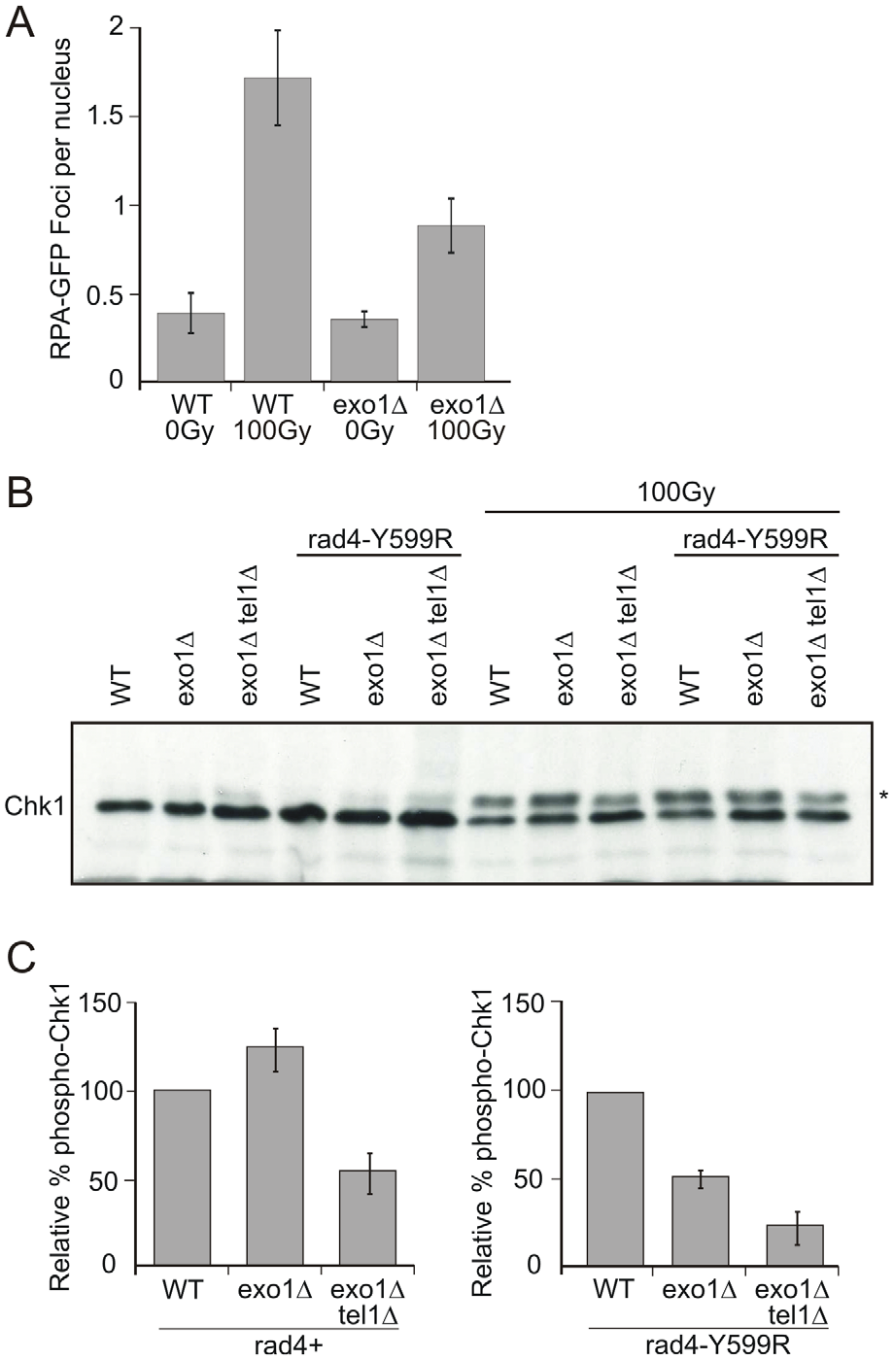
Chk1 phosphorylation is reduced in *rad4*-Y599R mutant cells when *exo1* is deleted. A. *exo1* ^+^ (WT) and exo1 deleted cells harbouring a *rad11^RPA^-GFP* allele were assayed for the number of Rad11^RPA^ foci either with no treatment or after 100 Gy ionising radiation. B. Chk1 phosphorylation (*) was monitored using α-HA following 100 Gy IR in the indicated strains (immediately after irradiation). C. The phosphorylated (reduced mobility) bands were quantified relative to unphosphorylated Chk1 for each strain indicated. Error bars are the standard deviation from the mean (n = 3).

To test this prediction we examined the induction of Chk1 phosphorylation in response to 100 Gy IR in *exo1*Δ *rad4* ^+^ and exo1Δ *rad4-Y599R* cells (Figure 6). First we established that, when *exo1* was deleted, Rad11^RPA^ foci were reduced in number, consistent with the expectationt hat resection is decreased in this background (Figure 6A). In contrast to *rad4* ^+^ cells, where *exo1* deletion did not reduce Chk1 phosphorylation levels, Chk1 phosphorylation was reduced to approximately 50% when *exo1* was deleted in AAD-defective cells. Previous work in both budding and fission yeasts has indicated that, in the absence of resection, Chk1 phosphorylation can occur through an alternative double strand break end-dependent Tel1^ATM^ pathway, as opposed to the canonical resection and ssDNA/RPA-dependent Rad3^ATR^ pathway [52]. Such a response could potentially mask some aspect of defects seen in the *exo1*Δ background. Thus, we first confirmed that loss of *tel1* alone does not influence Chk1 phosphorylation in our assay (Figure S1H) and then concomitantly deleted Tel1^ATM^ in both *rad4* ^+^ and *rad4-Y599R* strains (Figure 6B, 6C). In the *tel1*Δ exo1Δ background, Chk1 phosphorylation was decreased by approximately 50% for both *rad4* ^+^ and *rad4-Y599R* when compared to the *exo1*Δ alone background. These data are consistent with a general increase in Tel1-dependent checkpoint signalling when resection is reduced by *exo1* deletion. In the background of the *rad4-Y599R* mutation, this is superimposed on a decrease in Rad3^ATR^-dependent signalling caused by the reduced resection.

### The Rad4^TopBP1^ and Rad9 AADs co-operate in G1/S

**Figure 7:**
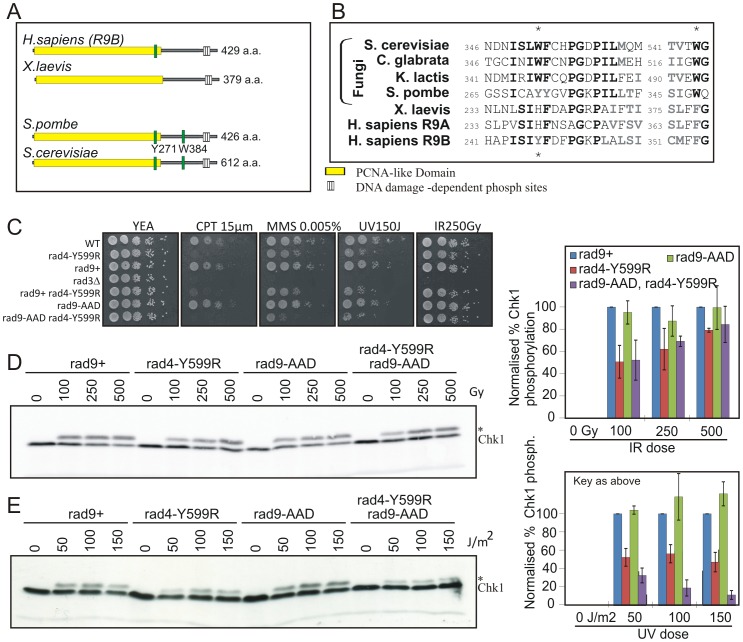
Analysis of the Rad9-AAD. A. Schematic representation of Rad9 homologs. The PCNA-like domain is boxed. The position of the conserved AAD aromatic residues are indicated in green. B. Corresponding amino acid sequence alignment. Conserved residues are in bold. The two aromatic residues are starred. C. Spot test analysis of sensitivity of the indicated strains to genotoxic treatments indicated. *rad9* ^+^ indicates the RMCE control strain [56]. D. Western blot analysis of Chk1-HA, detected with α-HA, in response the indicated dose of IR in *rad9* ^+^, *rad9-AAD*, *rad4*-Y599R and *rad9-AAD rad4-Y599R* double mutants. * indicates phospho Chk1. Quantification of the blot is given on the right. E. An equivalent experiment for the indicated doses of UV. Experiments for D and E have been reproduced three times and the normalised quantifications are shown graphically on the right. Error bars: standard deviation of the mean.

A second ATR activation domain has recently been identified in the *S. cerevisiae* Ddc1^Rad9^ C-terminal tail [26]. Mutations in this domain define a function in Mec1^ATR^ activation during G1, complementary to the function of the Dpb11^TopBP1^ AAD in promoting robust checkpoint activation in G2 in this organism [24]. Sequence alignments show that the two key Ddc1^Rad9^ AAD aromatic residues are conserved in *S. pombe* as Y271 (equating to W352^Sc^ within the PCNA-like domain) and W348 (equating to W544^Sc^ in the intrinsically disordered C-terminal tail) ([26] and Figure 7A, 7B).We thus created an *rad9-AAD* mutant by mutating both aromatic residues to alanine.

Analysis of the resulting *rad9-AAD* strain demonstrates no clear sensitivity to DNA damaging agents that create problems during S phase, including CPT, MMS and UV (Figure 7C) or in G2 to IR. However, some increased sensitivity is evident to CPT and MMS when the Rad4^TopBP1^ AAD mutant is present in the same strain. We next assayed the ability of *rad9-AAD* mutants to activate Chk1 in response to either IR or UV treatment. Consistent with the lack of sensitivity, the level of Chk1 phosphorylation after IR was not reduced (Figure 7D), either in *rad9-AAD* mutant alone or in the *rad9-AAD rad4-Y599R* double mutant when compared to the *rad4-Y599R* single. In response to UV treatment, there was again no decrease observed for the single *rad9-AAD* mutant, but a further and reproducible decrease was seen for the *rad9-AAD rad4-Y559R* double mutant when compared to *rad4-Y599R* alone (Figure 7E). Thus, the putative Rad9-AAD domain in *S. pombe* plays, at most, only a minor role in activating Rad3^ATR^ in response to DNA damage and this is only revealed in the absence of the Rad4^TopBP1^ AAD.

## Discussion

Understanding the mechanism of ATR activation is an important facet of gaining insight into how cells respond to unwanted DNA structures, itself a key aspect in maintaining genomic integrity. TopBP1 was initially implicated in the ATR-dependent checkpoint in fission yeast and later this was extended to higher eukaryotes [53]. TopBP1 is a multi-BRCT-domain containing protein that acts to scaffold proteins during both the initiation of DNA replication and in response to DNA damage, a function dependent of the phospho-binding ability of the BRCT-domain pairs within TopBP1 [25]. In addition to scaffolding phospho-proteins, TopBP1 was shown to be able to directly activate ATR in Xenopus and human cells through a small domain of TopBP1 which is not part of any BRCT pair [17]. The ATR activating domain is sufficient, both in vitro and in vivo, to activate ATR - although it is not always necessary for ATR activation and the pathway in which this TopBP1 AAD domain functions is yet to be fully understood. It has recently been shown in *S. cerevisiae* that the AAD of the TopBP1 homolog (Dpb11) is also able to activate the ATR homolg (Mec1). However, the Dpb11 AAD plays a relatively minor role in checkpoint activation which is specific to G2 phase [24]. In *S. cerevisiae*, a second ATR activation domain within the C-terminal tail of the 9-1-1 subunit, Ddc1^Rad9^ acts to help activate ATR in G1 and G2 and it is only when the function of this AAD is ablated a role for the Dpb11 AAD becomes apparent [26]. However, loss of both domains does not prevent checkpoint activation entirely, suggesting other AADs or modes of activation. Conversely, in the Xenopus system, ATR activation via the TopBP1 AAD is evident in S phase.

Here we show that, in *S. pombe*, the activation of the ATR homolog (Rad3) by a Rad4^TopBP1^ AAD is conserved. We demonstrate that the Rad4^TopBP1^ AAD makes a contribution to checkpoint activation and that this is specific to G1/S phase and is not evident in G2. Note that log phase *S. pombe* spend little, if any, time in G1 and thus, while we can arrest cells before the onset of replication with cell cycle mutants, we cannot make a clear physiological distinction between G1 and S phase. We go on to demonstrate that, when DNA resection was limited in G2 by ablation of the Exo1 nuclease, checkpoint activation in response to DNA damage during G2 becomes partially dependent on the Rad4^TopBP1^ AAD, mimicking what we observed in G1/S cells. This leads us to propose that there is a threshold of ssDNA required for activation of the DNA damage checkpoint and that the Rad4^TopBP1^ AAD serves to amplify checkpoint signals when ssDNA is limiting.

**Figure 8:**
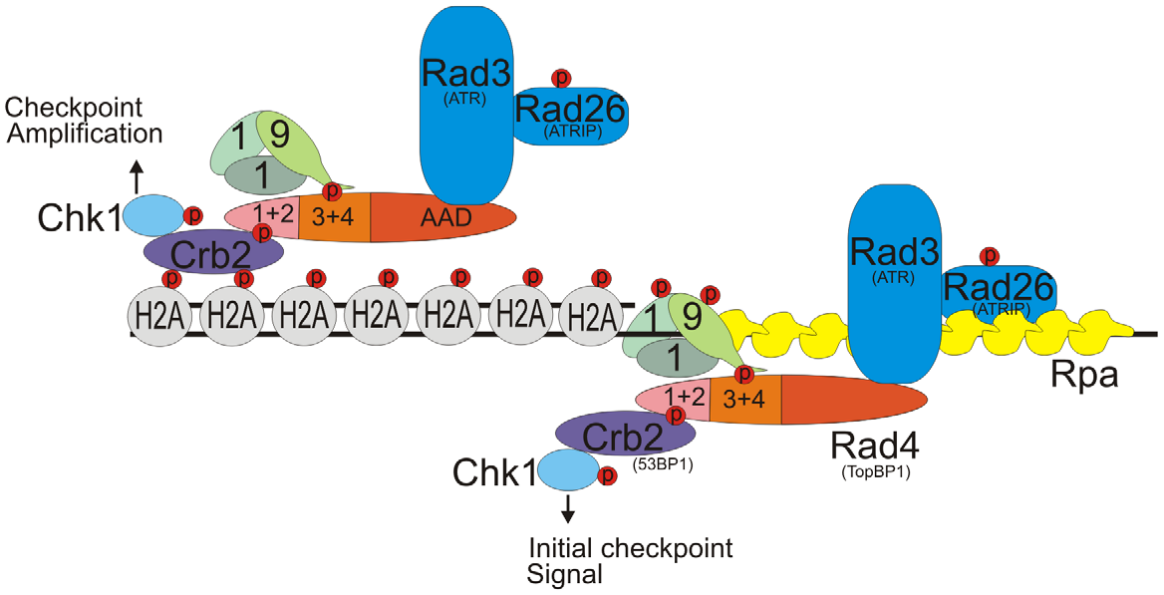
Updated model for the checkpoint response in *S. pombe*. Rad3^ATR^ is initially activated via an ssDNA pathway which is independent of the Rad4^TopBP1^ AAD (right side). However, a secondary pathway of Rad3^ATR^ activation, which is dependent on γH2A, its interaction with Crb2^53BP1^ and requires the Rad4^TopBP1^ AAD domain is important to amplify levels of activated Rad3^ATR^ and thus Chk1(left side). This chromatin Rad4-AAD pathway is of particular importance when the levels of ssDNA are low.

We next used a genetic system to separate Rad3^ATR^ activation from the production of DNA damage and therefore ssDNA, thus allowing us to assess the pathway of Rad3^ATR^ activation dependent on the Rad4^TopBP1^ AAD. In this system, specific checkpoint proteins are recruited to a defined chromatin locus through dsDNA:protein binding [37], [39]. Interestingly, recruitment of any one of the three checkpoint proteins (Rad3^ATR^, Rad4^TopBP1^ and Rad9) tested was sufficient to generate a checkpoint response and these responses followed the expected dependencies. This suggests that the recruitment of multiple copies of a single checkpoint protein results in the formation of active checkpoint complexes that utilise the endogenous proteins. Using this system, we observed that the ability of the Rad4^TopBP1^ AAD to activate Rad3^ATR^ is fully dependent on phosphorylation of H2A (γH2A) and requires the ability of Crb2 to bind γH2A. This leads us to conclude that, in the absence of ssDNA, the ATR activation domain of Rad4^TopBP1^ is particularly important for Rad3^ATR^ activation and acts in a chromatin:protein interaction dependent manner. Taking these data together with the requirement of the Rad4^TopBP1^ AAD to amplify checkpoint signals in either G1/S or G2 when resection was limited, we propose that Rad4^TopBP1^ acts to amplify the checkpoint in a chromatin-dependent manner when single-stranded DNA levels are limiting. We can therefore hypothesise that there is a threshold level for the amount of active Rad3^ATR^ required for a full checkpoint response. When ss-DNA is limited, such as in S-phase, the chromatin-dependent Rad4^TopBP1^ AAD-dependent pathway for Rad3^ATR^ activation becomes important to amplify the levels of activated Rad3^ATR^ to obtain a full checkpoint response (Figure 8).

In addition to analysing the Rad4^TopBP1^ AAD, we also created a mutant predicted to disable the Rad9 equivalent of the *S. cerevisiae* Ddc1^Rad9^ AAD and analysed the effect of this mutant in checkpoint activation. Unlike in *S. cerevisiae*, we observed no significant effect on DNA damage-induced checkpoint activation either in G1/S phase or G2. Although when combined with a Rad4^TopBP1^ AAD mutant, an additive effect to S-phase but not G2 DNA damage can be seen. This suggests that the Rad9 AAD acts in a separate but redundant pathway for Rad3^ATR^ activation in G1/S with the Rad4^TopBP1^ AAD. It appears that, during evolution, the mechanism of activating the ATR pathway has diverged significantly with the roles of different ATR activating domains being of more or less importance in different organisms.

It will be interesting to establish if the ATR activating domain of TopBP1 in metazoan systems is particularly important in the context of low levels of ssDNA and whether its function is dependent on γH2AX, especially as a 53BP1(Crb2) and TopBP1 pathway for checkpoint activation in G1 has been previously reported in the mammalian system [54]. The differences in the dependencies of the specific ATR activators in different cell cycle phases between *S. pombe* and *S. cerevisiae* is not surprising as the checkpoint mechanism between these organisms has diverged. For example, in *S. cerevisiae*, the S phase checkpoint is activated independently of the 9-1-1 complex, whereas in *S. pombe* and mammalian cells ATR activation appears to be largely - if not entirely - dependent on 9-1-1 loading. Such distinctions are likely a result of evolutionary adaptation to the different cell cycle profiles of the two yeasts and it is interesting to note that significant evolutionary plasticity surrounds the interface between TopBP1 and the checkpoint apparatus. These distinctions will have to be considered when extrapolating mechanistic data from yeast to human systems. None the less, we believe that our findings shed light on the role of TopBP1 AAD in DNA damage responses and offer useful insights into metazoan mechanisms of DNA damage signalling.

## Materials and Methods

### *S. pombe* strain construction and biological methods

Standard *S. pombe* protocols were carried out as previously described [55]. *rad4* and *rad9* mutant strains were created using PCR site directed mutagenesis and integrated at their endogenous locus using Cre recombinase-mediated cassette exchange [56] In brief, this system uses a “base strain” which is engineered so that the gene of interest is either replaced with the *ura4* marker (i.e. *rad9*), or in the case of essential genes (i.e. *rad4*) has the marker integrated immediately after the stop codon. In both cases the gene/marker and loci's promoter region are flanked by loxP and loxM sites. These two variant lox sites are incompatible with each other. The marker (and, for essential genes, the actual gene also) is then replaced by transforming in either the wild type (as a control: *rad* ^+^) or the various mutated copies on a plasmid. These are flanked by the equivalent *loxP* and *loxM* sites and the plasmid expresses Cre recombinase, which results in loxP:loxP and loxM:loxM recombination. For *cdc10-M17* synchronisation cells were grown to log phase at the permissive temperature (25°C) and shifted to the restrictive temperature of 36°C for 3.5 hours. Cells were then either irradiated with the indicated dose of gamma irradiation at 36°C and released at 25°C, or directly released at 25°C and irradiated at the given time points after release. *cdc25-22* block and release [57] and lactose gradient synchronisation [12] were performed as described previously. For FACS analysis cells were resuspended in 50 mM tri-sodium citrate, 1 mg/ml final concentration RNAseA [Sigma], stained with 5 µg/ml Propidium iodide [Sigma] and analysed on FacsCalibur [Becton Dickinson].

### Imaging

For live cell imaging concentrated culture was mounted onto a 2.5% agar patch in standard YE medim [Microworks] and imaged on a Deltavsion Microscope. Septation index was counted as previously described [12]. *lacO::NAT chk1-HA* strains were created by inserting the 10 Kb lacO repeats into the PUC19 plasmid containing the NAT marker and homology to *ura4*. This was integrated into the genomic *ura4* locus. The appropriate strains were transformed [58] with pRep41-GFP-LacI-NLS (GFP/LN) into which either *rad3* or *rad9* had been cloned in frame for N-terminal tagging or *rad4* cloned in frame for C-terminal tagging. Transformants were grown and expression of the fusion protein induced by the removal of thiamine. All *lacO* repeats were checked by Southern hybridisation.

### Biochemical assays

Protein extracts for western were prepared by TCA (trichloro-acetic acid) extraction from 1×10^8^ cells and resuspended in SDS sample buffer [59]. Crude extracts for affinity analysis were prepared by mechanical disruption in liquid nitrogen. Antibodies used: α-HA [Santa cruz] 1∶2500, α-Myc [Santa cruz] 1∶2000, α-GFP [Roche] 1∶2500, α-H2ApS129 [Abcam] 1∶2500 or 1∶1000, α-Tubulin [Sigma] 1∶5000, α-Cdc2 Sc-53 [Santa cruz] 1∶2500. α-Cdc13 [Jacky Hayles] 1∶500. α-Cds1 1∶5000 [35]. The secondary antibodies used were Hrp rabbit α mouse [Dako] 1∶2500 or Hrp swine α Rabbit [Dako]1∶2500. Chk1-HA phosphorylation was quantified as a percentage of total signal minus back ground on a ImageQuant LAS 4000 [GE Healthcare]. Cds1 kinase assay was carried out as described [35].

## Supporting Information

Figure S1 A. Sensitivity of the indicated strains to HU. 10-fold serial dilutions of 1×10^7^ cells/ml were spotted onto YEA. *rad4* ^+^ indicates the RMCE control strain [56]. B. Rad22-GFP foci were visualised by fluorescence microscopy before and after 40 Gy ionising radiation: average of 2 experiments. C-E. pREP41-GFP/LN fused to wild-type *rad3*, *rad3* kinase dead (*rad3*_KD_) *rad9* or *rad4* were used to transform wild type (WT) or appropriate mutant strains. Empty vector served as a control. Expression of the fusion proteins was induced (thiamine withdrawal for 16 hours) and 10-fold serial dilutions of 1×10^7^ cell/ml spotted onto selective media plates without thiamine either with or without HU or UV irradiation at the indicated doses. F. Rad3-GFP/LN (+R3) or an empty vector control (+EV) was induced for 22 hours in either *rad4* ^+^, *rad4-Y599R* or *brc1*Δ cells harbouring the *lacO* array. Rad3-GFP/LN expression was monitored 0 and 22 hrs after induction. Chk1-HA phosphorylation was used as an indicator of Rad3-dependent checkpoint activation. G. Rad3^ATR^ and Rad4^TopBP1^, when co-expressed as LacI fusions and co-recruited to a *lacO* array can bypass the requirement for Rad9. Top panel: *rad9* is required when Rad3 is recruited alone. Bottom two panels: two independent experiments showing co-recruitment bypasses *rad9* and remains *lacO*-dependent. H. Deletion of *tel1* alone does not affect Chk1 phosphorylation. Quantification, relative to unphosphorylated Chk1, of phosphorylated Chk1 following 100 Gy IR in the indicated strains immediately after irradiation (α-HA). Error bars are the standard deviation from the mean (n = 3).
(TIF)
