# Supplementary figures and images for: The Rad4TopBP1 ATR-Activation Domain Functions in G1/S Phase in a Chromatin-Dependent Manner
Source: PLoS Genet. 2012 Jun 28;8(6):e1002801. doi: 10.1371/journal.pgen.1002801 (PMC3386226; doi:10.1371/journal.pgen.1002801)

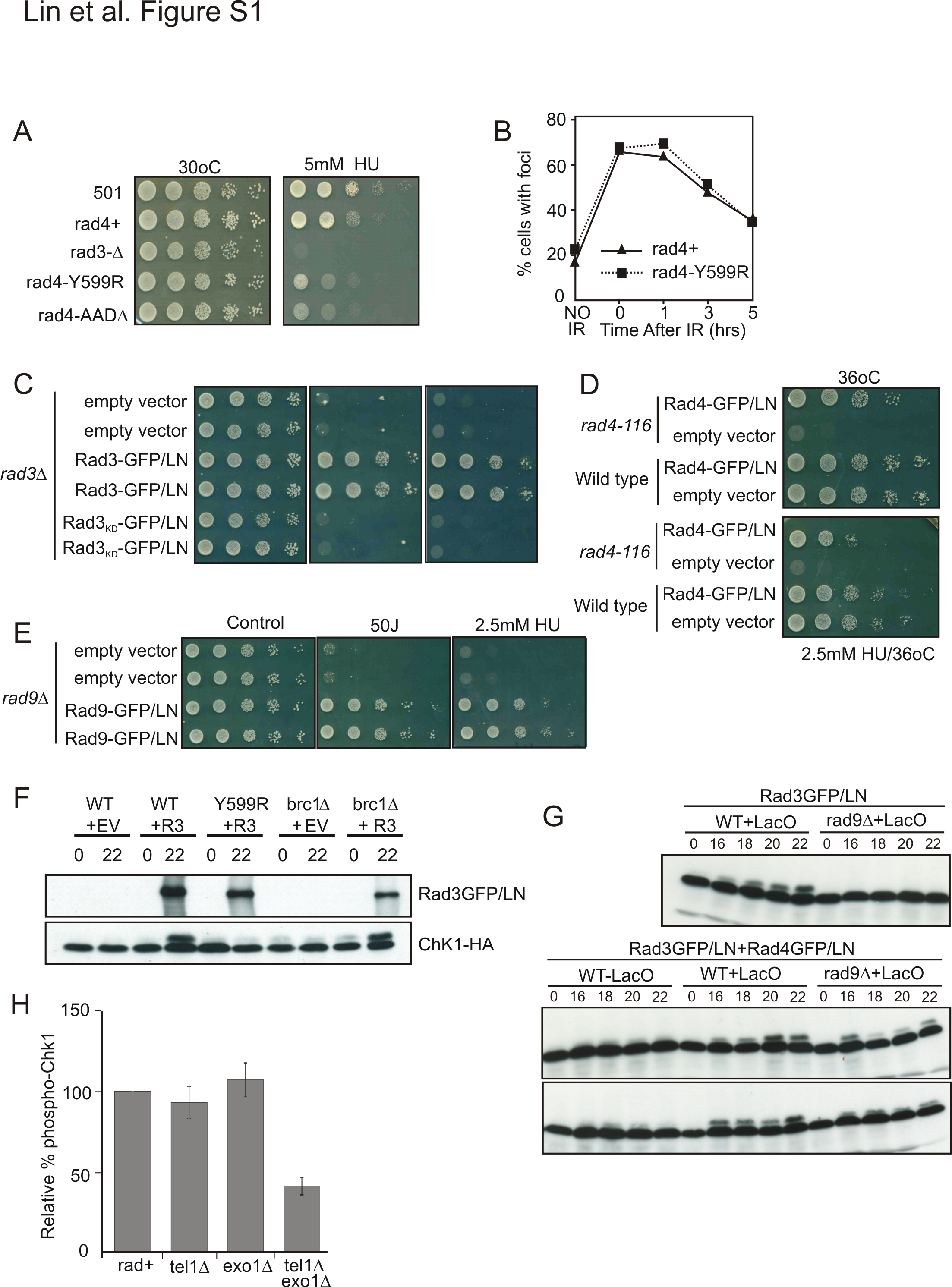

Supplement: Figure S1 — A. Sensitivity of the indicated strains to HU. 10-fold serial dilutions of 1×10^7^ cells/ml were spotted onto YEA. rad4 + indicates the RMCE control strain [56]. B. Rad22-GFP foci were visualised by fluorescence microscopy before and after 40 Gy ionising radiation: average of 2 experiments. C-E. pREP41-GFP/LN fused to wild-type rad3, rad3 kinase dead (rad3 KD) rad9 or rad4 were used to transform wild type (WT) or appropriate mutant strains. Empty vector served as a control. Expression of the fusion proteins was induced (thiamine withdrawal for 16 hours) and 10-fold serial dilutions of 1×10^7^ cell/ml spotted onto selective media plates without thiamine either with or without HU or UV irradiation at the indicated doses. F. Rad3-GFP/LN (+R3) or an empty vector control (+EV) was induced for 22 hours in either rad4 +, rad4-Y599R or brc1Δ cells harbouring the lacO array. Rad3-GFP/LN expression was monitored 0 and 22 hrs after induction. Chk1-HA phosphorylation was used as an indicator of Rad3-dependent checkpoint activation. G. Rad3ATR and Rad4TopBP1, when co-expressed as LacI fusions and co-recruited to a lacO array can bypass the requirement for Rad9. Top panel: rad9 is required when Rad3 is recruited alone. Bottom two panels: two independent experiments showing co-recruitment bypasses rad9 and remains lacO-dependent. H. Deletion of tel1 alone does not affect Chk1 phosphorylation. Quantification, relative to unphosphorylated Chk1, of phosphorylated Chk1 following 100 Gy IR in the indicated strains immediately after irradiation (α-HA). Error bars are the standard deviation from the mean (n = 3). (TIF) [file pgen.1002801.s001.tif]
